# Clinical Investigation of Recurrence, Oncological, and Obstetrical Outcomes in Patients with Ovarian Atypical Endometriosis

**DOI:** 10.3390/jcm14165656

**Published:** 2025-08-10

**Authors:** Su Hyeon Choi, So Hyun Shim, Seyeon Won, Nara Lee, Mi Kyoung Kim, Bo Wook Kim, Yong Wook Jung, Seok Ju Seong, Songmi Noh, Mi-La Kim

**Affiliations:** 1 Department of Obstetrics and Gynecology, CHA Gangnam Medical Center, CHA University, Seoul 06135, Republic of Korea; k345@chamc.co.kr (S.H.C.); simuso@chamc.co.kr (S.H.S.); drtong85@chamc.co.kr (S.W.); naradd@chamc.co.kr (N.L.); ra13811@chamc.co.kr (M.K.K.); kimbw@chamc.co.kr (B.W.K.); dumbung@chamc.co.kr (Y.W.J.); sjseong@cha.ac.kr (S.J.S.); 2 Department of Pathology, CHA Gangnam Medical Center, CHA University, Seoul 06135, Republic of Korea; npine@cha.ac.kr

**Keywords:** atypical endometriosis, recurrence, risk factor, endometriosis-associated ovarian cancer, in vitro fertilization

## Abstract

**Objectives**: The objective of this study was to evaluate the safety of postoperative in vitro fertilization (IVF) for atypical endometriosis (AE) in terms of ovarian endometrioma recurrence and development of endometriosis-related ovarian cancer (EAOC). **Methods**: Premenopausal women with AE who had undergone ovarian surgery between 2008 and 2022 and had attended follow-up appointments for at least 3 months were included in this retrospective study. The recurrence of endometriosis, postoperative pregnancy rate, occurrence of postoperative EAOC in cases of AE, and independent risk factors of AE recurrence were analyzed. **Results**: A total of 105 patients were included in the study with a median age of 33 years (range, 16–50 years) and a median follow-up duration of 29.0 months (range, 3–143 months). Most of the patients were treated with cyst enucleation (96.2%). Recurrent ovarian endometrioma was detected in 19 patients (18.1%), 4 of whom (19.0%) underwent reoperation, and there were no cases of EAOC. The cumulative recurrence rate at 12, 24, and 50 months was 7.4, 15.8, and 26.3%, respectively. Among the 105 patients, 36 wanted to become pregnant; of these, 12 underwent IVF, which, according to a univariable analysis, did not increase their risk of recurrent ovarian endometrioma. According to a subsequent multivariable analysis, previous history of ovarian endometrioma operation was the sole significant risk factor for AE recurrence (HR, 4.246; 95% CI, 1.262–14.285; *p* = 0.020). **Conclusions**: IVF trials for pregnancy did not represent a risk factor for recurrence, as treated AE showed a low possibility of malignant transformation, and IVF was not a risk factor for recurrence.

## 1. Introduction

Endometriosis is a benign disease characterized by the presence of endometrial glands and stroma outside of the uterus [1]. In approximately 0.5~1% of endometriosis cases, these tissues undergo malignant transformation [2,3]. Notably, atypical endometriosis (AE), characterized by cytologic atypia and architectural hyperplasia, is a known precursor lesion of endometriosis-associated ovarian cancer (EAOC) [4,5,6,7].

It is suggested that the clinical association between endometriosis and infertility is related to multifactorial processes, such as pelvic adhesions and chronic pelvic inflammation, that interfere with the processes of ovulation, oocyte uptake, sperm transport and function, gamete fertilization, and embryo migration and implantation. Assisted reproductive technology (ART) procedures can mitigate some of these adverse phenomena by addressing a spectrum of infertility problems, including ovulation disorders, fertilization failure, and damaged fallopian tubes [8]. Thus, there has been an increase in studies on whether it is safe to perform IVF in patients with endometriosis.

In a meta-analysis by Somigliana et al., in vitro fertilization (IVF) did not increase the risk of endometriosis recurrence [9]. In addition, Vassard et al. reported that ovarian cancer risk was increased among women with endometriosis who had undergone treatment with ART (HR, 3.78, 95% CI, 2.45–5.84) [10]. In a previous study, we found that AE has a higher recurrence rate than typical endometriosis [11]. However, there is limited evidence of IVF’s safety in cases of AE with regard to the risk of its recurrence or the occurrence of EAOC. Atypical endometriosis is a rare histopathologic diagnosis, with its reported incidence ranging from approximately <1% to 5.8% among patients with endometriosis in the absence of malignancy [12]. Due to its rarity, large-scale studies focusing specifically on AE remain scarce, so even studies with a relatively small number of AE cases can offer clinically meaningful insights. Therefore, the aims of the present study were to evaluate, in cases of AE, the recurrence rate, postoperative pregnancy rate, and risk factors (e.g., IVF trials) for recurrence, in addition to the occurrence of postoperative EAOC.

## 2. Materials and Methods

This retrospective cohort study covered the period between January 2008 and December 2022 and took place at a single gynecological surgery center. During this period, a total of 5312 patients underwent surgery for histologically confirmed ovarian endometriosis. Among them, 132 patients (2.48%) were pathologically diagnosed with atypical endometriosis (AE) based on central pathological review. Cases showing reactive atypia, regenerative atypia, or non-atypical hyperplasia were excluded to ensure diagnostic specificity. These 132 AE cases constituted the study cohort. Patients were excluded if they had undergone hysterectomy or bilateral oophorectomy (n = 5), had attended follow-up appointments for less than 3 months (n = 15), or had an associated ovarian malignancy (n = 7). The remaining 105 patients were premenopausal with no residual ovarian lesions, as confirmed by the first postoperative ultrasonography. This study was approved by the Institutional Review Board and ethics committee of CHA Gangnam Medical Center on the Use of Human Subjects in Research (GCI 2023-09-010-001), and informed consent requirements were waived given its retrospective nature. Additionally, the entire study was conducted in accordance with the ethical standards of the institution as well as the 1964 Declaration of Helsinki and its later amendments.

We reviewed and collected baseline data from patients’ medical charts, including age at surgery, parity, body mass index (BMI), size of ovarian cyst (largest diameter [cm]; if the ovarian cysts were bilateral, the sum of the largest diameters was recorded), duration of follow-up, preoperative cancer antigen 125 (CA 125) levels, preoperative anti-Müllerian hormone (AMH) levels, previous history of endometrioma operation, laterality of ovarian cyst(s), cyst nature (unilocular vs. multilocular), cul-de-sac (CDS) obliteration, revised American Society of Reproductive Medicine (rASRM) stage, operation method (cyst enucleation or unilateral oophorectomy), duration of postoperative hormonal treatment, subsequent pregnancy, mode of pregnancy (including use of IVF method[s]), time to recurrence, and, in cases of reoperation, prevalence of EAOC. The duration of postoperative medication was calculated using the sum of individual medications used (i.e., gonadotrophin-releasing hormone agonist (GnRH agonist), oral progestin including dienogest (DNG), oral contraceptive pills (OCPs), or levonorgestrel–intrauterine system (LNG-IUS)) before confirmed recurrence.

**Figure 1:**
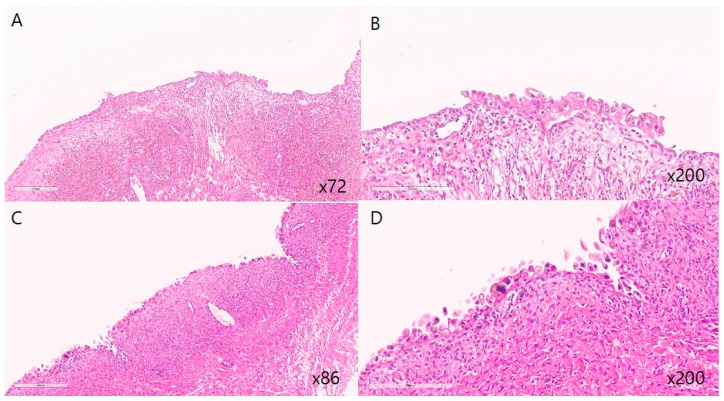
Representative histopathologic features of atypical endometriosis. (**A**,**C**) Endometriotic cyst showing attenuated endometrial lining with sparse stroma (low-power view. (**B**,**D**) Epithelial cell atypia characterized by sporadic nuclear enlargement and hyperchromasia with preservation of low N/C ratio in endometriotic cyst (high-power view). Cytologic atypia with nuclear enlargement and hyperchromasia.

**Figure 2:**
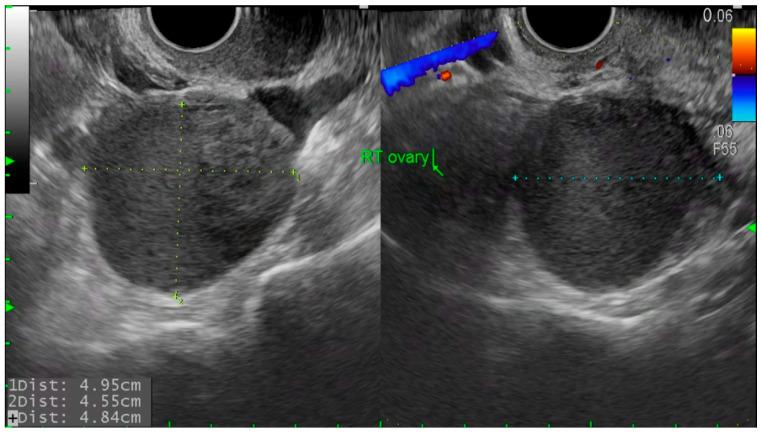
Transvaginal ultrasound image of recurrent ovarian endometrioma. Round, thick-walled cyst with homogenous low echogenic content and scattered internal echoes, measuring over 2 cm in diameter.

At our institution, initial gynecological examination and transvaginal ultrasonography (TVS) or transrectal ultrasonography (TRS) were performed 3–6 months postoperatively, and if the reports were unremarkable, follow-ups were performed at intervals of 6–12 months. Additional follow-ups were performed if the patient developed any symptoms. During every follow-up visit, patients were asked to fill out pain scales and specify their marital, pregnancy, and childbirth status. According to the patients’ pregnancy plan and status, we categorized them as No plan/Failure to conceive/Successful delivery/Miscarriage or ectopic pregnancy/Ongoing pregnancy or lost to follow-up (unknown pregnancy outcome after confirmed intrauterine pregnancy). Time to recurrence was defined as the time in months from the surgery to detection of a newly developed ovarian endometrioma measuring 2 cm or more. Recurrence of ovarian endometrioma was defined when TVS or TRS showed the following findings: a round cystic mass of ≥20 mm with thick walls, irregular margins, homogenous low-echogenic fluid content, scattered internal echoes, or negative papillary proliferation [13]. The representative images used for diagnosis are provided in Figure 1 (histologic images of atypical endometriosis) and Figure 2 (ultrasound images of recurrent endometrioma). If a patient had two endometriomas each measuring <20 mm, the diameters were added to obtain a value ≥ 20 mm, and this measurement was used to define recurrence [14,15]. Statistical analysis was performed using SPSS 25.0 software (SPSS Chicago, IL, USA).

Between the recurrent (n = 19) and non-recurrent groups (n = 86), categorical variables were compared using the Chi-squared or Fisher’s exact test. Quantitative variables were compared using the Mann–Whitney U test after the normality of the data was determined using the Shapiro–Wilk test. The Kaplan–Meier method was used to calculate the cumulative probability of recurrence. A multivariable analysis including the significant variables from the univariable analysis was performed using Cox’s proportional hazards model to obtain a subset of independent risk factors for recurrence of ovarian endometrioma. Among these variables, those with a *p*-value < 0.2 were subjected to multivariable regression analyses. *p* values < 0.05 were considered statistically significant.

## 3. Results

### 3.1. Patient Characteristics

#### Baseline Clinical Features

Table 1 shows the baseline characteristics of the 105 AE patients recruited in this study. The median age was 33 years (range, 16–50 years), and 75 (71.4%) patients were nulliparous. The median follow-up duration was 29.0 months (range, 3–143 months). Most of the patients were treated with cyst enucleation (96.2%).

### 3.2. Recurrence Outcomes

#### 3.2.1. Recurrence and Reoperation

Recurrent ovarian endometrioma was detected in 19 patients (18.1%), 4 of whom (19.0%) underwent reoperation. There was no EAOC in the reoperation cases.

#### 3.2.2. Cumulative Recurrence Rates

**Figure 3:**
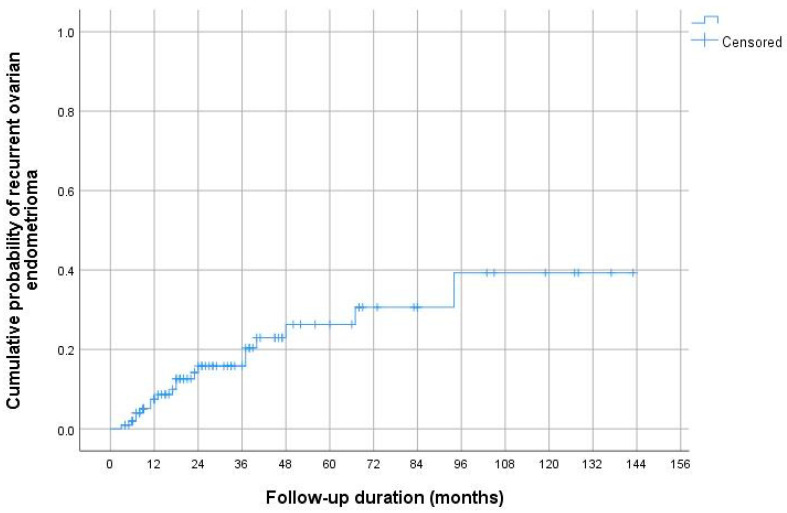
Cumulative ovarian endometrioma recurrence rates according to Kaplan–Meier analysis. The cumulative AE recurrence rates 12, 24, and 60 months after surgical treatment were 7.4, 15.8, and 26.3%, respectively.

Using the Kaplan–Meier method, the cumulative AE recurrence rates 12, 24, and 60 months after surgical treatment were 7.4, 15.8, and 26.3%, respectively (Figure 3).

### 3.3. Fertility Outcomes and Risk Factor Analysis

#### 3.3.1. Pregnancy and IVF Outcomes

Among the 36 patients who wanted to become pregnant after their operations, 25 (69.4%) conceived, 16 (44.4%) of whom had successful deliveries. In addition, 12 (48%) of the 36 patients who wanted to become pregnant underwent an IVF trial, and 2 conceived naturally after failed IVF.

#### 3.3.2. Risk Factors for Recurrence: Group Comparison

The results of the analysis of risk factors for recurrence of ovarian endometriosis are listed in Table 2. The recurrent group had a history of endometrioma operation (*p* = 0.005), CDS obliteration (*p* = 0.022), and rASRM stage IV (*p* = 0.003). However, IVF trials for pregnancy did not increase the risk of recurrent ovarian endometrioma (*p* = 0.227).

#### 3.3.3. Risk Factors for Recurrence: Cox Regression Analysis

The results of the univariable and multivariable Cox regression analyses of independent risk factors for recurrent ovarian endometrioma are shown in Table 3. According to the univariable analysis, previous history of ovarian endometrioma (HR, 5.833; 95% CI, 1.986–17.127; *p* = 0.001), complete CDS obliteration (HR, 4.121; 95% CI, 1.307–12.994; *p* = 0.016), and rASRM stage IV (HR, 4.138; 95% CI, 1.370–12.494; *p* = 0.012) were significant risk factors for recurrence of AE. In this analysis, once again, IVF trials for pregnancy were not associated with risk factors for recurrence (HR, 2.033; 95% CI, 0.659–6.268; *p* = 0.217). According to the subsequent multivariable analysis, previous history of ovarian endometrioma operation was the sole significant risk factor for recurrence of AE (HR, 4.246; 95% CI, 1.262–14.285; *p* = 0.020).

## 4. Discussion

The main finding of this study was that IVF trials in AE patients did not increase the risk of endometriosis recurrence, whereas a previous history of ovarian endometrioma operation was found to be a risk factor for recurrence. Postoperative EAOC did not occur in any of the cases of AE investigated. During the 40.9 ± 34.8-month follow-up period, no patients were diagnosed with malignant transformation. To the best of our knowledge, this is the first cohort study to evaluate AE recurrence after IVF trials.

Endometriosis is regarded as a definitive risk factor for ovarian cancer, the most fatal gynecological cancer [2]. Malignant transformation of endometriosis is considered a rare occurrence, affecting approximately 0.5~1% of ovarian endometriomas [2,3]. Large-cohort and case–control studies indicate an increased risk of ovarian cancer in endometriosis patients [16,17,18,19,20,21], with some documenting that endometriosis is associated with an approximately 3-fold statistically significant increase in the risk of endometrioid and clear-cell ovarian cancer [4,16,19,21,22,23,24,25]. Cyclic hemorrhage of endometriotic cysts leads to the accumulation of blood components and can induce inflammation through oxidative stress, which potentiates DNA damage [26]. Additional molecular alterations have also been noted, including ARIDA1/BAF250a, PIK3CA, CTNNB1, and PTEN mutation; microsatellite instability; and loss of heterozygosity [27,28,29,30,31].

Atypical endometriosis (AE) is known to be precancerous and strongly related to endometriosis-associated ovarian cancer (EAOC), characterized by cytologic atypia and architectural atypia or hyperplasia [4,7]. It is estimated that approximately 60 to 80% of all EAOC cases occur in the presence of AE, which is often directly related to tumors [32]. Numerous studies have suggested that AE might be a middle or transitional step in progression from benign to cancerous disease [33].

Meanwhile, there have been many concerns about whether IVF increases ovarian cancer. Two theories supporting the association between fertility drugs and ovarian cancer have been proposed, namely, the ‘incessant ovulation theory’ and the ‘elevated gonadotrophin theory’ [34]. The first suggests that repetitive damage to the epithelial surface of the ovary and subsequent DNA repair cycles contribute to the risk of ovarian cancer [35,36], while the second claims that exposure to high levels of circulating gonadotrophins stimulates the epithelium on the ovarian surface and increases the risk of DNA damage [37]. Other studies have reported no association of repeated ART cycles with ovarian cancer [38,39,40].

In a much more recent investigation, the risk of ovarian cancer after ART treatment differed substantially depending on the cause of infertility [10]. It was not increased in women with female-factor infertility (excluding endometriosis) or in women referred for unexplained or male-factor infertility; in contrast, in ART-treated women with endometriosis, the risk of ovarian cancer was increased 3.78-fold. These results support the hypothesis that ovarian cancer is more likely associated with the characteristics of individual women than with ART-treatment factors such as endometriosis, ART parameters such as ovarian stimulation, or ART procedures [10].

Somiglinana et al. investigated the impact of IVF procedures on endometriosis, endometriotic lesions, and endometriosis-related pain and recurrence [9]. IVF did not worsen endometriosis-related pain symptoms or increase the risk of endometriosis recurrence, and its impact on ovarian endometriomas was non-existent or mild [9]. Likewise, in the present study, although a relatively small number of patients were analyzed, we found that IVF procedures in cases of AE did not increase the rate of endometriosis recurrence or occurrence of EAOC.

Furthermore, to address concerns regarding reproductive and obstetric outcomes, we analyzed the clinical characteristics of the 25 AE patients who achieved pregnancy, including their IVF history, pregnancy outcomes, and recurrence status (Table 4). Among these 25 patients, 6 experienced recurrence, and of these 6 patients, 4 of delivered. Of these four patients, two developed recurrence after delivery, while the other two developed recurrence prior to pregnancy. The latter two patients delivered via cesarean section at different hospitals, and it is unclear whether ovarian surgery was performed concurrently. Although our study did not analyze delivery mode outcomes in detail, we suggest that vaginal delivery should be performed if there are no obstetric indications. In cases requiring cesarean section and where recurrent ovarian lesions are accessible and symptomatic, concurrent surgical management may be considered.

While these findings provide meaningful preliminary evidence, they warrant careful interpretation. Only 12 patients in our cohort underwent IVF, and this limited sample size increases the possibility of a type II statistical error. Because of the retrospective design and the rarity of pathologically confirmed AE, a priori sample size calculation was not feasible, which may have limited the statistical power of our analyses. Furthermore, as this was a single-center observational study, causal relationships could not be established, and subgroup analysis based on IVF success or pregnancy outcomes was not possible.

Our study has several additional limitations. First, we did not consider different types of hormonal therapy. We analyzed only recurrence rates, based on the durations of combinations of hormonal treatments. Second, we defined recurrence as the presence of cysts larger than 20 mm as identified by ultrasonography, rather than by histological confirmation. Third, due to the retrospective nature of the study, it was not possible to conduct detailed comparisons of the IVF protocols or drugs employed, or the numbers of IVF procedures. Finally, in the group of patients who became pregnant after IVF treatment, the patient number was too small and the follow-up duration was relatively short. Nevertheless, the strength of this investigation lies in the fact that it is the first cohort study to analyze the impact of IVF trials on AE recurrence or malignant transformation.

In a previous study, we found that AE has a higher recurrence rate than typical endometriosis [10]. In the present study, approximately 18.1% of patients diagnosed with AE experienced recurrent ovarian endometrioma; however, there was no occurrence of EAOC, and IVF was not a risk factor for recurrence. Considering that treated AE showed a low possibility of malignant transformation, and that IVF was not a risk factor for recurrence, we conclude that it is safe for women who want to become pregnant to undergo IVF procedures under surveillance. However, to confirm our findings, further large-scale prospective studies are needed.

## Figures and Tables

**Table 1:** Baseline characteristics of atypical endometriosis (AE) patients (n = 105).

Baseline Characteristics	Mean ± SD (Median, Range)/n (%)
Age at surgery (years) ^1^	34.0 ± 6.3 (33, 16–50)
Parity ^2^	
Nulliparous	75 (71.4%)
Parous	30 (28.6%)
Body mass index (kg/m^2^) ^1^	21.4 ± 4.1 (20.3, 15.1–47.2)
Size of ovarian cyst(s) (cm) ^1^	8.0 ± 3.9 (6.9, 3.0–26.0)
Duration of follow-up (months) ^1^	40.9 ± 34.8 (29, 3–143)
Preoperative CA125 (n = 93) ^1^	67.8 ± 72.6 (44.8, 3.6–575.7)
Preoperative AMH (n = 67) ^1^	3.24 ± 2.68 (2.81, 0.11–10.19)
Previous history of endometrioma operation ^2^	
No	97 (92.4%)
Yes	8 (7.6%)
Laterality ^2^	
Unilateral	72 (68.6%)
Bilateral	33 (31.4%)
Cyst nature ^2^	
Unilocular	62 (59.0%)
Multilocular	43 (41.0%)
Cul-de-sac obliteration ^3^	
None	40 (38.1%)
Partial	32 (30.5%)
Complete	33 (31.4%)
rASRM stage ^2^	
Stage III	54 (51.4%)
Stage IV	51 (48.6%)
Operation method ^2^	
Ovarian cystectomy	101 (96.2%)
Oophorectomy	4 (3.8%)
Surgical approach	
Conventional laparoscopy	82 (78.1%)
Robot-assisted laparoscopy	21 (20.0%)
Open laparotomy	2 (1.9%)
Medication duration ^3^	
None	21 (20.0%)
<6 Mo	12 (11.4%)
≥6–<12 Mo	16 (15.2%)
≥12–<24 Mo	29 (27.6%)
≥24 Mo	27 (25.7%)
Duration of postoperative hormonal treatment (months) ^1^	19.3 ± 24.4 (12, 0–143)
Subsequent pregnancy ^3^	
No plan for pregnancy	69 (65.7%)
No pregnancy	11 (10.5%)
Confirmed pregnancy	25 (23.8%)
Mode of pregnancy (n = 25) ^2^	
Natural	13 (52%)
IVF	12 (48%)
Recurrence after previous operation ^2^	19 (18.1%)
Time to recurrence (months) (n = 19) ^1^	25.8 ± 23.6 (18, 3–94)

Data are presented as mean ± standard deviation (median, range) or number (%). CA125, cancer antigen 125; AMH, anti-Mullerian hormone; rASRM, revised American Society of Reproductive Medicine; IVF, in vitro fertilization. ^1^ Mann–Whitney U test. ^2^ Fisher’s exact test. ^3^ Chi-squared test.

**Table 2:** Analysis of risk factors for recurrence of ovarian endometriosis (n = 105).

Characteristics	No Recurrence (n = 86)	Recurrence (n = 19)	*p*-Value
Age at surgery (years) ^1^	34.0 ± 6.6 (33, 16–50)	33.7 ± 4.8 (33, 26–43)	0.967
Parity ^2^			0.269
Nulliparous	63 (73.3%)	12 (63.2%)	
Parous	23 (26.7%)	7 (36.8%)	
Body mass index (kg/m^2^) ^1^	21.6 ± 4.3 (20.4, 15.1–47.2)	20.7 ± 9.0 (20.3, 17.0–29.3)	0.403
Size of ovarian cyst(s) (cm) ^1^	8.0 ± 3.7 (7.0, 3.6–26.0)	8.1 ± 4.8 (6.0, 3.0–23.6)	0.699
Duration of follow-up (months) ^1^	36.5 ± 33.3 (25, 4–143)	63.3 ± 34.3 (54, 20–128)	0.001
Preoperative CA125 (n = 93) ^1^	65.4 ± 76.0 (43.9, 3.6–575.7)	79.5 ± 54.2 (70.1, 15.7–203.2)	0.171
Preoperative AMH (n = 67) ^1^	3.27 ± 2.73 (2.71, 0.11–10.19)	3.07 ± 2.42 (2.95, 0.42–7.08)	0.956
Previous history of endometrioma operation^2^			0.005
No	83 (96.5%)	14 (73.7%)	
Yes	3 (3.5%)	5 (26.3%)	
Laterality ^2^			0.379
Unilateral	60 (69.8%)	12 (63.2%)	
Bilateral	26 (30.2%)	7 (36.8%)	
Cyst nature ^2^			0.353
Unilocular	52 (60.5%)	10 (52.6%)	
Multilocular	34 (39.5%)	9 (47.4%)	
Cul-de-sac obliteration ^3^			0.022
None	36 (41.9%)	4 (21.1%)	
Partial	28 (32.5%)	4 (21.1%)	
Complete	22 (25.6%)	11(57.8%)	
rASRM stage ^2^			0.003
Stage III	50 (58.1%)	4 (21.1%)	
Stage IV	36 (41.9%)	15 (78.9%)	
Operation method ^2^			0.444
Ovarian cystectomy	82 (95.3%)	19 (100%)	
Oophorectomy	4 (4.7%)	0 (0%)	
Medication duration ^3^			0.772
None	17 (19.8%)	4 (21.1%)	
<6 Mo	10 (11.6%)	2 (10.5%)	
≥6–<12 Mo	14 (16.3%)	2 (10.5%)	
≥12–<24 Mo	25 (29.1%)	4 (21.1%)	
≥24 Mo	20 (23.2%)	7 (36.8%)	
Duration of postoperative hormonal treatment (months) ^1^	18.4 ± 24.6 (12, 0–143)	23.2 ± 23.4 (20, 0–88)	0.308
Subsequent pregnancy ^3^			0.403
No plan for pregnancy	59 (68.6%)	10 (52.6%)	
No pregnancy	8 (9.3%)	3 (15.8%)	
Confirmed pregnancy	19 (22.1%)	6 (31.6%)	
Mode of pregnancy (n = 25) ^2^			0.555
Natural	12 (63.2%)	3 (50.0%)	
IVF	7 (36.8%)	3 (50.0%)	
IVF trial for pregnancy (including no plan for pregnancy) ^2^			0.227
No	76 (88.4%)	15 (78.9%)	
Yes	10 (11.6%)	4 (21.1%)	

Data are presented as mean ± standard deviation (median, range) or number (%). CA125, cancer antigen 125; AMH, anti-Mullerian hormone; rASRM, revised American Society of Reproductive Medicine; IVF, in vitro fertilization. ^1^ Mann–Whitney U test. ^2^ Fisher’s exact test. ^3^ Chi-squared test.

**Table 3:** Univariable and multivariable Cox regression analyses of independent risk factors for recurrence of ovarian endometrioma.

Risk Factors for Recurrence	Univariable HR (95% CI)	*p*-Value	Multivariable HR (95% CI)	*p*-Value
Age	0.996 (0.924–1.074)	0.923		
Parous (vs. nulliparous)	1.354 (0.529–3.468)	0.527		
BMI	0.966 (0.847–1.101)	0.603		
Size of ovarian cyst(s)	1.024 (0.915–1.146)	0.680		
Preoperative CA125	1.001 (0.997–1.006)	0.586		
Preoperative AMH	0.955 (0.748–1.219)	0.710		
Previous endo op (vs. no previous op)	5.833 (1.986–17.127)	0.001	4.449 (1.368–14.472)	0.013
Bilateral (vs. unilateral)	1.002 (0.393–2.555)	0.997		
Multilocular (vs. unilocular)	2.152 (0.850–5.447)	0.106	1.757 (0.629–4.911)	0.283
CDS obliteration		0.014		0.728
None	1.0		1.0	
Partial	1.096 (0.273–4.401)	0.898	0.564 (0.137–2.327)	0.429
Complete	4.121 (1.307–12.994)	0.016	0.702 (0.162–3.035)	0.635
rASRM stage IV (vs. III)	4.138 (1.370–12.494)	0.012	2.135 (0.524–8.698)	0.290
Oophorectomy (vs. cystectomy)	21.177 (0.000–6960792.275)	0.638	4.449 (1.368–14.472)	0.013
Medication duration		0.910		
None	1.0			
<6 Mo	0.609 (0.110–3.384)	0.571		
≥6–<12 Mo	1.079 (0.194–5.995)	0.930		
≥12–<24 Mo	0.589 (0.147–2.363)	0.456		
≥24 Mo	0.652 (0.185–2.293)	0.505		
Duration of postoperative hormonal treatment	0.993 (0.977–1.010)	0.430		
Subsequent pregnancy		0.407		
No plan for pregnancy	1.0			
No pregnancy	2.275 (0.622–8.327)	0.214		
Confirmed pregnancy	1.548 (0.560–4.275)	0.399		
Postop pregnancy (vs. no postop pregnancy)	1.350 (0.511–3.567)	0.545		
IVF trial for pregnancy (vs. no IVF trial)	2.033 (0.659–6.268)	0.217		

HR, hazard ratio; CI, confidence interval; BMI, body mass index; CA125, cancer antigen 125; AMH, anti-Mullerian hormone; endo, endometrioma; op, operation; CDS, cul-de-sac; rASRM, revised American Society of Reproductive Medicine; Mo, month; IVF, in vitro fertilization.

**Table 4:** Clinical characteristics and pregnancy outcomes of patients with atypical endometriosis who achieved pregnancy during follow-up.

No.	Age at Surgery (Yr)	Parity	Tumor Size (cm)	Surgical Treatment	Subsequent Treatment	Time to Pregnancy (Months)	Pregnancy Methods	Delivery Weeks	Pregnancy Outcome	Recurrent Disease	Time to Recurrence (Months)	Recurrent Operation
1	34	0	4.7	L/S LOCE	No	34	IVF		Ongoing pregnancy	X		
2	31	0	6	L/S LOCE	GnRHa	90	Natural		Ongoing pregnancy	O	94	
3	31	0	5	L/S LOCE	GnRHa	8	Natural		NSVD	X		
4	36	1	5.7	L/S ROCE	No	3	IVF		Ongoing pregnancy	X		
5	33	0	7.4/4.6	L/S BOCE	No	17	Natural		Ongoing pregnancy	X		
6	32	1	4.5	L/S LOCE	GnRHa	84	Natural		AA	O	24	L/S BOCE
7	40	1	6.9	L/S LOCE	No	1	Natural	Full-term	C/S	X		
8	30	0	8.8	L/S ROCE	No	2	IVF	38 + 6	C/S	X		
9	36	0	3	L/S LOCE	No	13	IVF	39 + 2	C/S	O	37	
10	29	0	6.5	L/S LOCE	GnRHa	21	Natural	39 + 6	NSVD	X		
11	33	0	8.4/5.8	L/S BOCE	No	7 38	IVF Natural	Full-term 38 + 2	NSVD NSVD	X		
12	30	0	6.2	Robotic LOCE	No	8 26	IVF Natural	Full-term Full-term	C/S C/S	X		
13	28	0	5.4	L/S ROCE	No	19 58	Natural, Natural	35 + 6, 36 + 6	PSVD, PSVD	X		
14	32	0	4.2	L/S ROCE	No	3	Natural	40	NSVD	X		
15	30	0	10.5/1.7	L/S BOCE	No	2	Natural		Abortion	X		
16	30	1	7.9	L/S ROCE	No	13	Natural	38 + 2	C/S	X		
17	34	0	6.1/1.3	L/S ROCE, LOC aspiration	No	17	IVF		Ongoing pregnancy	X		
18	34		5	L/S LOCE	GnRHa	26	Natural	39 + 2	C/S	X		
19	32	1	5.2	L/S LOCE	No	3	Natural	Full-term	C/S	X		
20	34	0	4.1	Robotic ROCE	GnRHa	12	IVF	38	C/S	X		
21	33	0	6.2	L/S LOCE	No	17	Natural	Full-term	C/S	X		
22	32	0	6.6/5.4	L/S BOCE	No	7	Natural	37 + 3	C/S	O	48	L/S BOCE
23	31	0	5.2	L/S LOCE	No	8	No data		Abortion	X		
24	32	0	8.1/6.6	L/S BOCE	No	43	IVF	Full-term	C/S	O	7	
25	27	0	5.9/2	L/S BOCE	GnRHa	40	IVF	Full-term	C/S	O	23	

L/S, laparoscopic; LOCE, left ovarian cyst enucleation; IVF, in vitro fertilization; GnRHa, gonadotropin-releasing hormone agonist; NSVD, normal spontaneous vaginal delivery; ROCE, right ovarian cyst enucleation; BOCE, bilateral ovarian cyst enucleation; AA, artificial abortion; C/S, cesarean section; PSVD, premature spontaneous vaginal delivery; LOC, left ovarian cyst.

## Data Availability

The data that support the findings of this study are available from the corresponding author upon reasonable request.
